# ‘Developmental Delay’ Reconsidered: The Critical Role of Age-Dependent, Co-variant Development

**DOI:** 10.3389/fpsyg.2018.00503

**Published:** 2018-04-23

**Authors:** Yonata Levy

**Affiliations:** Department of Psychology, Hebrew University of Jerusalem, Jerusalem, Israel

**Keywords:** developmental co-variance, temporal dependency, cortical maturation, gene expression, language delay

## Abstract

***In memory of Annette Karmiloff-Smith***.

This paper reviews recent neurobiological research reporting structural co-variance and temporal dependencies in age-dependent gene expression, parameters of cortical maturation, long range connectivity and interaction of the biological network with the environment. This research suggests that age by size trajectories of brain structures relate to functional properties more than absolute sizes. In line with these findings, recent behavioral studies of typically developing children whose language development was delayed reported long term consequences of such delays. As for neurodevelopmental disorders, disrupted developmental timing and slow acquisitional pace are hallmarks of these populations. It is argued that these behavioral and neuro-biological results highlight the need to commit to a developmental model which will reflect the fact that temporal dependencies overseeing structural co-variance among developmental components are major regulatory factors of typical development of the brain/mind network. Consequently, the concept of ‘developmental delay’ in developmental theorizing needs to be reconsidered.

## Introduction

In research as well as in developmental clinics, development is looked upon in reference to three potential states: typical, delayed or deviant. This paper considers recent behavioral and neurobiological research, suggesting that the juxtaposition between delay and deviance underplays the significance of temporally dependent, co-variant development. Temporal dependency refers to the fact that two or more brain areas or behavioral components mature at the same chronological age, whereas by developmental co-variance we refer to the fact that development proceeds along similar structural parameters.

The importance of chronological age rests on the premise that age is correlated with brain maturation and environmental interaction. In atypical development, however, age is not a reliable predictor of brain maturation. Given the nature of neurodevelopment disorders, it is to be expected that temporal dependencies and structural covariance will be disrupted. Area specific brain maturation may be slower (e.g., Wolff et al., 2012), or faster (e.g., Weinstein et al., 2011), or it may take an atypical course, which in some cases may nevertheless result in typical performance (e.g., Annaz et al., 2009). Genetic irregularities, disorders of timed gene expression and mis-aligned environment are liable to disrupt the biological clock and hence the correlation between age and maturation.

A focus on the mind/brain as an interconnected network suggests the critical role of equilibrium among components and consequently the importance of temporal dependencies and developmental co-variance in brain development as well as in behavioral domains. From this perspective, ‘developmental delay’ requires a new conceptualization, which will consider age as an independent variable, differentially reflected in temporal and structural dependencies among functional components, as well as in interaction with the environment. Based on the data reviewed in this article, it will be argued that developmental theorizing requires a commitment to an integrated developmental model, whose major regulatory principle is a reflection of the fact that temporal dependencies and structural covariance characterize typical development.

An example of the problematic nature of the concept of ‘developmental delay’ and the insufficient consideration of the potential consequences of developmental a-synchrony is evident in the way developmental delay has been applied in pediatrics. The term ‘developmental delay’ as it is used in the clinic is a descriptive term, not a diagnosis. It is meant to describe children whose performance resembles that of a younger child, as distinct from children who show patterns of performance not shown in typically developing (TD) children as well as children who have received a clear diagnosis. Whereas performance of children with developmental delay indeed resembles performance of children of a younger age, it is often the case that children with diagnosed disorders likewise show within domain performance that characterizes typical developmental trajectories (e.g., Paterson et al., 1999; Brock, 2007; Levy and Eilam, 2013). Importantly, the term ‘developmental delay’ is reserved to children under 5 years, signaling, as the term connotes, conditions that could be transient. In practice, however, most children with developmental delays continue to show developmental deficits into late childhood and even adulthood.

Developmental delay can be global or specific. Global developmental delay (GDD) is defined in reference to infants and preschoolers, ages 0–5 years, who present with delays of 6 months or more, in two or more of the following developmental domains: gross/fine motor, speech/language, cognition, social/personal and daily living activities. GDD is typically seen in 1–3% of children and is “a symptom complex with heterogeneous presentations, causes, associated conditions and evolution over time” (Riou et al., 2009, p. 600). A similar definition appears in DSM-V (American Psychiatric Association, 2013). Specific developmental delay (SDD) refers to age inappropriate performance in a specific area. Prevalence of SDD varies with the area concerned.

These facts have been acknowledged by the professional community and problems with the use of the term ‘delay,’ in particular in what concerns the implication that the phenomena are likely short-lived, have been repeatedly pointed out. A substitute term, ‘Early Developmental Impairment,’ was suggested with the implication that as the child matures, an established diagnosis was likely (Francouer et al., 2010). In practice, however, the term GDD continues to be widely used, as its lack of specificity qualifies it as a ‘place holder’ in the communication between clinician and parents, as well as in discourse within the professional community (Shevell, 2010). As for SDD, Gillberg (2010) pointed out difficulties in defining the domain of concern in a young child, along with the fact that in most cases deficits in additional domains are likely to emerge later, typically resulting in claims about “comorbidity” among syndromes. It is argued that what is referred to as comorbidity is in fact a reflection of the interdependence between parts of the developing brain network, that are gradually unfolding as the child grows up.

An important concept, highlighting the role of chronological age in the interaction between structural/functional brain development and the environment is that of critical, or sensitive, periods that has been around for three decades (Greenough et al., 1987). Sensitive periods refer to the age ranges during which the system is especially sensitive to specific experiences, which, in turn, impact structural and functional changes. Such changes are less likely to take place at other periods in development (Knudsen, 2004). Different explanations have been offered for the observed reduction in brain plasticity that characterizes the termination of sensitive periods (Thomas and Johnson, 2008). Importantly, sensitive periods refer to specific segments of the developmental course during which interaction between developmental factors is optimal, whereas recent research reviewed in the current paper suggests that timing plays a vital role across domains and along the entire developmental trajectory.

Significant research has considered the impact of environmental deprivation on missed sensitive periods and the potential for long term effects of environmental deficits (e.g., Rymer, 1993; Nelson et al., 2014). Almas et al. (2016) reported adverse effects of early psycho-social deprivation, specifically insecure attachment, on IQ at age 12. Musical training of just 1 year in early childhood can alter motor, language and auditory areas (Schneider et al., 2002; Gaser and Schlaug, 2003). Using a population modeling approach Thomas and Knowland (2014) found that variation among simulated individuals with language delays was caused by differences in internal neurocomputational learning parameters as well as the nature of the language environment. The developmental trajectories suggested that richness of the language environment did not predict the emergence of persisting language delay, but did predict the final ability levels of individuals whose language delay was resolved by age 4–5.

The impact of learning and training on brain morphology is not restricted to sensitive periods. Thus, learning to read at all ages affects gray matter density in language areas (Carreiras et al., 2009; Dehaene et al., 2010) and expert taxi drivers benefit from larger hippocampi volume relative to their work experience (Maguire et al., 2003). It is still not known to what extent these experiences affect co-variance among brain areas.

Disorders of timing, i.e., delays of onset, slow progress and premature halt, are a hallmark of neurodevelopmental disorders. An attempt to maintain age as a reference point when evaluating a child’s performance is the concept of mental age (MA) as it applies to these populations. MA in fact offers a substitute “age” for the study of disordered populations. Importantly, cognitive performance indexed by MA is typically attributed to intellectual potential, often at the cost of neglecting other age-related factors affecting cognitive development. Thus, in studies of populations with cognitive impairments participants are regularly compared to much younger controls, who, although matched on MA, differ on a variety of variables such as experience with age-appropriate environment, emotional development, social interactions and physical development. In an effort to further localize the deficit and hence the choice of controls, concepts such as language-age, or face processing age were introduced referring to performance in the area of interest, as distinct from the general cognitive functioning of the child (e.g., Rice, 2012 and see the discussion in Plante et al., 1993). Importantly, such concepts leave no room for considerations of the network properties of cognition, among them the critical role of temporal dependencies within the system, in particular in what concerns age-relevant environment. Thus, neither MA nor its domain-specific substitutes can be adequate proxies of chronological age.

Neuroconstructivism (Karmiloff-Smith, 1998; Karmiloff-Smith et al., 2012) is the developmental approach which places strong emphasis on structural dependencies within the brain network and the impact of the developmental process in interaction with the environment. Neuroconstructivism underlines the dynamics of development in cases of early brain disorders and the effect it may have on the network as a whole. Considering developmental trajectories of neurodevelopmental disorders, the claim has been that in the face of brain atypicalities, the resulting system could not be described “as a normal brain with parts intact and parts impaired, as the popular view holds” (Karmiloff-Smith et al., 2012, p. 393). It is suggested that the brain of a child with neurodevelopmental disorder be characterized as “an atypical system developing under different constraints” (D’Souza and Karmiloff-Smith, 2017, p. 4).

Following the seminal work by Karmilof-Smith, neuroconstructivism focused primarily on structural dissimilarities in the developmental course between disordered and typical populations (e.g., Karmiloff-Smith et al., 1997; Laing et al., 2002; Thomas and Karmiloff-Smith, 2003; Paterson et al., 2006). Importantly, however, there has been some concern with age and timing as well. In line with neuroconstructivist thinking, Morton and Frith (1995) offered a causal model of autism which considered the unfolding of the phenotype over developmental time. Morton and Frith emphasized the dynamic nature of the process of development yet they made no reference to chronological age. More recently, in a précis of neuroconstructivism, Sirois et al. (2008) referred to chronotropy, stressing that some patterns of gene expression occurred at specific developmental times, some aspects of neural development depended upon ordered events, and plasticity occurred at different ages in different parts of the developing system. In a similar vein, Thomas et al. (2009) stressed the fact that when developmental trajectories rather than data points were documented, age was not eliminated and could be considered in the comparison.

The approach advocated in the current review fully supports a neuroconstructivist approach to development, embracing its emphasis on the interdependence among components and the dynamic nature of development. Our aim is to underscore the critical role of age as a primary determinant of typical development. We draw attention to a recent body of research highlighting temporal dependencies and developmental co-variance among genetic/brain/environmental parameters. This research suggests that developmental schedule, i.e., chronological age, is a major parameter overseeing typicality. Ultimately, we argue, these data require a new conceptualization of the notion of ‘developmental delay,’ given that a missed schedule, specific or global, may impact the network as whole. Our approach echoes Rutter and Pickles’s (2016) view, who considered the neglect of the differential impact of age on environmental conditions and manipulations among the major threats to the validity of child psychology and psychiatry.

In the sections below results of state of the art neurobiological and behavioral studies underlining the central role of temporal dependencies and structural co-variance in typical and atypical populations are summarized. With respect to brain development, temporal dependencies and developmental co-variance were evident in brain morphology and gene expression in anatomically and/or functionally connected brain areas. The conclusion from these studies was that brain development was best characterized by measures of age-by-structure.

Is there evidence of long term effects of disorders of timing in behaviorally diagnosed conditions? As stated above, such correlations have been documented in pediatric clinics raising questions as to the use of the term ‘developmental delay’ in this context (Francouer et al., 2010; Gillberg, 2010). In view of the neurobiological evidence, presented below, a fresh look at the effects of disorders of timing is vital in the context of developmental research as well. A case in point concerns recent research in language development, summarized below, from which it follows that delay in language development is not cost free. Rather, developmental delays in language development have long term effects in typical as well as atypical populations.

## Age Related Effects and Covariant Developmental Changes Seen in Imaging Studies of Typical Populations

Age-related, temporal dependency is a major factor over-seeing long range connectivity and covariance of brain morphological features such as increased or decreased cortical volume (CV) or degree of gyrification. In a recent review chapter summarizing their work on a lifespan neurodevelopmental MRI database, Richards and Xie (2015) cite multiple studies stressing the problems in using adult reference MRIs for the study of brain development. The authors stress the fact that problems in this practice were not limited to infancy but were seen across the lifespan. Research suggested that brain variation across ages were likely to introduce spurious differences between the study group and the template. Brain networks that are anatomically connected have been shown to co-vary in their morphological features and these correlations resulted from similarities in timed maturational trajectories (Zielinski et al., 2010; Alexander-Bloch et al., 2013). For example, posterior and anterior language areas in the left hemisphere co-varied in their cortical thickness (Lerch et al., 2006). Similarly, motor, auditory and visual systems showed patterns of structural co-variance (Zielinski et al., 2010). Age related organizational changes in the brain networks measuring inter-regional correlations between 4 and 18 years, revealed a window of plasticity during late childhood, potentially subserving the developmental challenges facing emergent adulthood (Khundrakpam et al., 2013). Both synchronized rates of change and structural co-variance appear to be maximal in the association cortex (Raznahan et al., 2011a).

Recent research suggested that age by size trajectories of brain structures were related to functional properties more than absolute sizes (Giedd and Rapoport, 2010). Synchronized maturational change and structural covariance were seen for regions in the same functional modules, indicating that covariation in cortical anatomy coincided with cortical modular functional organization (Alexander-Bloch et al., 2013). Interestingly, similar to regions showing some form of physical connectivity, areas showing functional connectivity (seen through synchronous neuronal firing) but **no** anatomical connectivity, likewise showed strong morphological co-variance. Maturation of brain structural co-variance manifested age-dependent linear as well as non-linear progressions, likely reflecting functional distinctions between brain systems. Thus, the co-variant networks that tended to develop linearly included areas related to language and attention, whereas primary sensory and motor areas tended to expand more significantly at younger ages (Zielinski et al., 2010; Khundrakpam et al., 2013).

Recent imaging studies attempt to unpack critical components of structural parameters involved in connectivity among regions. In a longitudinal study of cortical thickness and cortical surface area in 647 individuals, ages 3–30 years, developmental changes in cortical thickness as well as in surface area were age-dependent and gender specific (Raznahan et al., 2011b; Giedd et al., 2015). Tamnes et al. (2010) studied age related changes in cortical thickness, regional white matter (WM) volume and diffusion characteristics. One hundred and sixty-eight healthy participants aged 8–30 years were scanned. The results showed regional age-related cortical thinning, WM volume increases and changes in diffusion parameters. All measures showed unique associations with age, yet cortical thickness was the most strongly age-related parameter. Importantly, age by cortical thickness was more predictive of IQ than differences in thickness alone (Shaw et al., 2006). Forde et al. (2017) investigated the effect of age and sex on cortical development in 218 healthy adolescents. Cortical thickness and local gyrification index, but not surface area, were inversely associated with age in all regions. An innovative curvature analysis (Intrinsic Curvature, IC, detailed in Forde et al., 2014) was likewise inversely associated with age in all but the occipital region.

A longitudinal study of TD babies, <1–12 months, revealed a maturational, non-linear developmental sequence of the brain networks. Network specific critical developmental periods were identified in the following order: primary sensorimotor/auditory, visual, attention/default mode and control/executive function (Gao et al., 2015). Langeslag et al. (2013) performed resting-state functional magnetic resonance imaging (Rs-fMRI) to determine whether parietal-frontal functional connectivity was associated with intelligence in young children. Results were age-related, suggesting that higher intelligence in children 6–8 years old was associated with increased connectivity of the right parietal region and the right frontal region. Association was stronger in girls than in boys.

In sum, results showed age-related structural changes in anatomically as well as in functionally connected brain networks. Age by size trajectories of cortical thickness, cortical surface area and WM maturational processes were temporally correlated across functional trajectories.

## Age Related Effects and Asynchronous Development Seen in Imaging Studies of Populations With Neurodevelopmental Disorders

In a review article, Courchesne et al. (2011) put forth a theory of age-specific anatomical abnormalities in autism spectrum disorder (ASD), potentially affecting the timed balance among brain networks. It was argued that an abnormally accelerated brain overgrowth was seen in children diagnosed with ASD between ages 2–4 years, followed by an abnormally slow or arrested growth between early and later childhood. An accelerated rate of decline in brain size was seen from adolescence to middle adulthood. Such an aberrant developmental schedule is likely to results in age-specific defects. Results reported in Hazlett et al. (2011) suggest that abnormal increase in CV seen in males with autism could result from derailing maturational timing of surface area rather than from cortical thickness. The developmental trajectory of cortical surface area predicted that detrimental effects were likely to occur in males rather than in females, and in the early years rather than later in development, thus supporting clinical observations in this population (Duvekot et al., 2017).

A recent study of brain WM in high-risk infant siblings of children with autism reported higher fractional anisotropy (FA) at 6 months, followed by slower changes over time in siblings who were later diagnosed with ASD, relative to siblings without ASD (Wolff et al., 2012). By 24 months, those with ASD had lower FA values in 12 out of 15 fiber tracts examined. In contrast with these results, Weinstein et al. (2011) reported increased FA in the left superior longitudinal fasciculus (left-SLF) and the body of the corpus callosum in 2–3 year old, low-functioning children with autism. It was suggested that increased FA reflected premature maturation of the left-SLF in autism, which could signal diminished plasticity for language.

A study of inter-regional covariance in children with ASD suggested a disruption of temporally regulated growth curve that was particularly evident in the social brain networks (McAlonan et al., 2005). In a similar vein, decreased covariance was reported between the amygdala and the fusiform gyrus, likely reflecting the diminished experience with faces in young children with ASD (Dziobek et al., 2010). A structural MRI study of children with ASD at two time points (Time point I: mean age = 4.1 years; Time point II: mean age 6.6 years) revealed a lack of age-related reduction of cortical thickness within areas related to language, social cognition and behavior control. Age-related gains in expressive language correlated with gains in cortical thickness in the right hemisphere homolog areas, likely the result of compensatory processes (Smith et al., 2016). Resting state fMRI (Rs-fMRI) showed age-related reduced connectivity between two higher-order cognitive networks in ASD and an interaction effect in the default mode network (DMN): insula connectivity increased with age in ASD, whereas it decreased with age in typically developing children (Bos et al., 2014).

As for children with attention deficit hyperactivity disorder (ADHD), developmental delays of trajectories of cortical thickness were noted specifically for the frontal lobes (Shaw et al., 2007). The median age by which 50% of the cortical points attained peak thickness was 7.5 years in TD children, and 10.5 years in children with ADHD. The greatest differences were seen in the middle prefrontal cortex, which reached peak thickness in typical children at 5.9 years, whereas it did not reach its maximum thickness until 10.9 years in children with ADHD. Clearly, the behavioral effects of ADHD were not a function of optimal cortical thickness in itself, but rather of the age at which the desired values were attained. Posner et al. (2014) review article of rs-fMRI studies of connectivity within DMN and the interactions between the DMN and the cognitive control network in people with ADHD supports the above MRI findings. Evidence for delayed neuro-maturation was seen as well (Posner et al., 2014). The neuropsychological correlates of these delays remain sparsely explored.

In sum, accelerated brain over-growth, disorders of WM anisotropy and disrupted development of the brain social network in children with autism were all age related. As for children with ADHD, although cortical thickness as well as connectivity within the DMN attained typical measures, development was slower than normal and did not follow typical schedule.

## Age Related Effects in Gene Expression and Neurobiological Processes in Typical Populations

A key aspect of age-sensitive effects orchestrating synchronous development among networks is the timing of gene expression. Changes in brain structures that emerge out of gene-environment interaction at preset timing include pruning of neurons and synapses and myelination of neuronal tracts, resulting in inhibition of sprouting and a reduction in plasticity, likely involved in the termination of the sensitive period (Fields, 2008). Heritability of cortical thickness is regional and age-related (Schmitt et al., 2014). Heritability effects were predominating in primary motor and sensory areas in younger children, whereas later maturing areas such as prefrontal cortex, superior temporal gyri and superior temporal lobe show increased heritability in older children and in adolescents, likely due to age-related gene expression (Lenroot et al., 2009). These results are in line with Plomin et al. (1997) longitudinal adoption study which showed age-related differences in the heritability of IQ.

The three-way interaction between age, genes and environment and the way this interdependence shapes developmental trajectories has been a focus of neurobiological research (Lenroot and Giedd, 2011). Minute disruptions of timing of synaptogenesis and pruning were seen to affect connectivity (Levitt, 2003). Studies in primates point to differences in the time course of histogenesis among functional areas. Consequently, environment will have a differential effect on neuronal development in accordance with the time needed to form the relevant connections (Rakic, 2006). Age-related effects of different polymorphisms of the brain-derived neurotrophic factor (BDNF) gene have mostly been studied in mice, but were also seen in humans (Casey et al., 2009). In mice as well as in human subjects, effects on hippocampal size as well as on memory and learning in response to stressful life events were pronounced during infancy but not during young adulthood. This is because infancy is low in BDNF whereas adulthood is high in BDNF. Furthermore, negative effects of low BDNF were restricted to those with BDNFmet allele (BDNF methionine), but were not evident in those with BDNFval allele (BDNF valine). For example, in post institutionalized children, 4–12 years old, who were adopted into families, those with BDNFmet allele, but not those with the val allele, had smaller cortical volumes relative to the non-institutionalized controls with the same allele (Choi et al., 2009).

## Age Related and A-Synchrony Effects of Gene Expression and Neurobiological Processes in Atypical Populations

Geschwind and Levitt (2007) stated what has become a truism in genetic research in general and in research on autism in particular, i.e., “In neurodevelopmental disorders, the timing, location and degree to which gene expression is disrupted dictate the emergent phenotype” (p. 103). In a recent review article de la Torre-Ubieta et al. (2016) citing Geschwind and Levitt (2007) and Abrahams and Geschwind (2010) state that “… even small changes in synaptic function and timing will preferentially disrupt the connectivity of higher-order association areas that mediate social behavior, which include the frontal-parietal, frontal-temporal and frontal striatal circuits. Identification of the spatiotemporal dynamics of transcriptional and translational regulation and the subsequent changes in micro- and macro-circuit connectivity will be necessary to link synaptic dysfunction to complex behavioral traits in individuals with ASD” (ibid, p. 354).

In a similar vein, the theory of age-related anatomical brain changes in autism hypothesized by Courchesne et al. (2011) was extended by these authors to gene expression as well. It was argued that in ASD, gene expression abnormalities in the adolescent and adult brain were not expected to be the same as those in the prenatal or toddler years and that delayed gene expression in the early years would have consequences for later development. Thus, results obtained with older children and adults were prone to reflect outcome associations rather than causal ones.

Differential effects of age were seen in the genetics of schizophrenia as well. Disruption of age dependent changes in the expression of schizophrenia susceptibility genes in the prefrontal cortex occurring during the critical period, were likely to dispose the individual to schizophrenia (Choi et al., 2009). Thus, Disrupted-In-SCizophrenia-1 (DISC-1) gene allelic effects affected maturational schedule of fronto-cortical areas, which, in turn, increased risk levels for specific schizophrenia disease phenotypes.

Finally, Kendler et al. (2008) reported genetic effects on symptoms of anxiety and depression, from middle childhood to young adulthood. The first genetic factor, which accounted for 72% of the variance in symptoms at ages 8–9, diminished in influence by ages 19–20, accounting for only 12% of the variance. New sets of genetic risk factors were emerging in adolescence and early adulthood.

In sum, timed gene expression is a key factor in processes related to pruning, myelination, brain plasticity and probably also the termination of sensitive periods in typical populations. Age related processes are seen in atypical populations as well. Age related effects of gene expression were hypothesized for ASD, while disruptions of timed gene expression were reported for schizophrenia, anxiety and depression.

Whereas studies pointing to temporal dependencies and developmental co-variance characterizing brain development abound, not many studies have considered developmental dependencies and synchrony among behavioral domains. Below we review studies in language development that relate to the long term effects of disorders of timing, mostly within the verbal domain.

## Behavioral Studies Reporting Long Term Impact of Developmental Language Delay

Language development is a research area in which the role of temporal dependencies and developmental co-variance have not been fully appreciated. A central example concerns toddlers who are late talkers (LTs) in the absence of any other condition which might be associated with language delay. LTs are estimated at 5–8% and are typically described as having a language delay, likely to resolve in three out of four children in the course of their preschool years (Bishop et al., 2012). According to the thesis of the current review, however, children with “resolved delay” are nevertheless likely to show long term deficits in the verbal modality and possibly also in other cognitive domains. Recent research supports this statement.

Late talkers with resolved delay consistently remain within the lower end of the normal distribution on most language measures at least into the 5th grade. These results were first reported by Paul (1996) and replicated in numerous subsequent studies, as summarized in a review article by Rescorla (2011), and more recently in a study by Rescorla and Turner (2015). Longitudinal studies of teenagers with a history of LTs showed significantly lower performance on grammaticality judgments as well as on tasks involving ambiguous sentences (Rescorla, 2011). Note that while judgments of grammaticality are in the verbal modality and although they revolve around knowledge of grammar, they engage meta-cognitive skills. Low performance on these tasks along with good oral language skills, suggest that it is not language *per se*, but the interaction of problem solving strategies and perhaps other domain relevant skills with language-related “riddles,” that creates insurmountable difficulties for adolescents with a history of LT.

Of relevance to the current discussion are children with specific language impairment (SLI). SLI is defined as a significant language delay in the absence of hearing loss, low level non-verbal intelligence, neurological conditions or other clinically defined impairments. Most (though not all) 4–5 year old children diagnosed with SLI were LTs. While the majority of children with SLI achieve adequate everyday language in the early school years, school age children with a history of SLI do not reach above 75% correct replies on judgments of grammaticality (Tomblin et al., 1997). A similar leveling was apparent in receptive vocabulary in adolescents with a history of SLI.

Difficulties in literacy skills have often been noted in children with a history of SLI, including children who did not differ from controls on vocabulary and comprehension (Stothard et al., 1998). Furthermore, advanced linguistic and meta-linguistic tasks and literacy skills were not the only tasks that children with SLI had deficits on. Durkin et al. (2013) examined numerical skills in children with SLI at age 7, and then again at age 8. Average scores were more than 1 SD below the population mean and language level correlated with number skills, highlighting the interdependence between domains. Recently, there has been an ongoing debate over the use of the term SLI, which has only too often been interpreted to mean that language is the only domain in which there are deficits. In view of the results reported above, we go along with Bishop (2014) and interpret ‘specific’ as ‘idiopathic.’ Finally, Rice (2012) offered to account for the deficits seen in children with SLI in reference to a dysfunctional timing mechanism responsible for the late onset and early leveling of language performance in this population. Rice argument remains a working hypothesis as to date there is no neurobiological evidence that supports it.

As for children with neurodevelopmental disorders, delays in the onset of language and slow acquisitional pace are the hallmark of these populations. Unlike patterns of performance in older children, studies of the early phases of language development suggested similarities in the order of emergence of grammatical structures, types of errors produced and mean utterance length across syndromes (e.g., Paterson et al., 1999; Levy and Eilam, 2013). Importantly, statistically significant, syndrome specific differences in age of onset of combinatorial language as well as in acquisitional pace were evident in the above studies. While timing was atypical in most populations with neurodevelopmental disorders, the extent of the delay, the acquisitional pace and the age at which a premature halt was observed differed among syndromes, suggesting a syndrome-specific biological basis for these aberrant developmental schedules (Levy and Eilam, 2013).

In sum, the data suggest that delays in language development in otherwise TD children have long-term consequences. Children with a history of SLI show long term deficits in the verbal and numerical modalities. Language delays characterize most neurodevelopmental disorders. Yet, the extent of the delay, as well as children’s ultimate achievements seem to be syndrome specific, suggesting a biological impact.

## Summary and Discussion

Current studies of brain development reviewed above highlight the role of temporal dependencies and developmental covariance between brain networks, suggesting that the distinction between delay and deviance, although useful in daily encounters with distressed parents in the clinic, can be misleading in the context of developmental theorizing. Especially problematic is the notion of SDD because, given the interdependent, structurally co-variant processes of brain development in the early years, it is unlikely that delay in achieving developmental milestones will be restricted to a specific area. In most cases time will uncover additional deficits. Thus, the common phenomenon referred to as ‘comorbidity’ among syndromes is more adequately conceptualized in reference to temporal dependencies and structural covariance among relevant components (see Gillberg, 2010). Among others, the behavioral study of ADHD as well as that of LTs and language delays, characteristic of a certain percentage of otherwise TD children as well as of many of the neurodevelopmental syndromes, could benefit from an approach which advocates comprehensive rather than specific evaluation, and an early rather than late intervention.

In view of the results presented in the current review, a re- conceptualization of developmental delay in behavioral studies is called for, which will consider age as an independent variable, differentially reflected in temporal and structural dependencies among functional components, as well as in interaction with the environment. It will bring to the fore a commitment to an integrated developmental model which will reflect the fact that temporal dependencies and structural covariance are major characteristics of typical development. A first step toward achieving this goal consists in defining a strategy for data integration, eventually enabling the construction of a unified developmental model. Aligning developmental trajectories of domains that develop in lockstep, with age as a variable, seeking to highlight temporal dependencies, would be a first step toward a consideration of the entire puzzle rather than pieces thereof. This a move is likely to highlight connections and dependencies that have not been uncovered thus far. Such an approach would be in line with a neuroconstructivist perspective on development and its emphasis on properties of the brain network, yet it will bring to the fore the central regulatory factor of development, i.e., chronological age.

Should we continue to distinguish between delay and disorder? The studies reviewed in the current paper suggest that as far as developmental theorizing is concerned, there is no room for this distinction, as delay foretells divergent developmental course, which, from a theoretical point of view, defies typicality. The issue is less clear with respect to the use of ‘delay’ in the clinic, where considerations involving the patient and her family are of primary concern. While most children with developmental delays will end up with a diagnosis, given brain plasticity and compensatory mechanisms, there would be cases in which delay will be resolved with no further complications. In fact, research has uncovered cases in which compensatory mechanisms in neurodevelopmental disorders may lead to adequate performance (See for example, Annaz et al., 2009 study of face-processing in children with autism, Down syndrome and Williams syndrome). Thus, one may argue that perhaps there is no harm in breaking the news to the parents piecemeal and in a gradual manner, as long as the professionals remain realistic and treatment is comprehensive. Alternatively, one can revert to the more accurate, yet still semi-benign phrase, and describe the child as having an ‘early developmental impairment,’ as suggested by Francouer et al. (2010).

Importantly, in view of the fact that the data are mostly correlational, the directionality of the effects cannot be established. At present, the causal relations between behavioral delay and the neurobiology of developmental disorders remain unknown. Does behavioral delay affect neuroanatomy, which then results in further behavioral deficits? Alternatively, given that there is some data showing that the extent of the delay is syndrome specific (Levy and Eilam, 2013), is this the result of a disrupted biological clock that is affected in ways that are unique to each syndrome? A related question concerns the very concept of a biological clock and its relevance to development in the early years. Note that in fact, a ‘biological clock’ could be the product of timed gene expression in interaction with a temporally adequate environment. If this is the case then disrupted developmental timing would be the external manifestation of failure of one or all factors involved in this interaction.

In sum, a temporal biological program regulating development, perhaps throughout the entire life cycle of an individual, must be incorporated into our theoretical accounts of typical and atypical neurodevelopment. A growing awareness of the effects of the circadian clock on human health provides further support for this claim (Roenneberg and Merrow, 2016). For example, there is evidence relating disorders of the circadian clock and dementia (Musiek, 2017). Note, however, that this is still work in progress and I am not aware of developmentally relevant research in this area. In the context of neurodevelopmental disorders, one can hypothesize that a congenital disorder of a ‘biological clock,’ resulting in a misalignment of developmental milestones as well as environmental effects, might have a detrimental impact on development.

## Author Contributions

The author confirms being the sole contributor of this work and approved it for publication.

## Conflict of Interest Statement

The author declares that the research was conducted in the absence of any commercial or financial relationships that could be construed as a potential conflict of interest.
